# Corrigendum

**DOI:** 10.1002/prp2.948

**Published:** 2022-05-05

In Li et al. (2022),^1^ the below sentence have been included in the acknowledgment section:

“We deeply appreciated the kindly gift of all the myricetin derivatives from Professor Wei Xue of Guizhou University.”

The updated acknowledgment section is given below:
